# Polarity dependent Hippo-Yap regulation controls cortical development

**DOI:** 10.21203/rs.3.rs-10644281/v1

**Published:** 2026-09-11

**Authors:** Seonhee Kim, Olivia Pericak, Raehee Park, Ambrosia Simmons, Johanna Dam, Justin Le, Noelle Sterling, Kathleen Rajavong, Casey Lim, Conchi Estaras, Seo-Hee Cho

**Affiliations:** Lewis Katz School of Medicine at Temple University; Lewis Katz School of Medicine at Temple University; Lewis Katz School of Medicine at Temple University; Lewis Katz School of Medicine at Temple University; Lewis Katz School of Medicine at Temple University; Lewis Katz School of Medicine at Temple University; Lewis Katz School of Medicine at Temple University; Lewis Katz School of Medicine at Temple University; Lewis Katz School of Medicine at Temple University; Lewis Katz School of Medicine at Temple University; Thomas Jefferson University

## Abstract

Epithelial polarity is established by partitioning the apical and basal domains within the cell. Antagonistic inhibitions between the apical and basal polarity complex proteins segregate them into their designated domains. Failure to achieve this causes developmental disorders and cancer. While the biochemical basis of this antagonism has been identified, the complexity of context-dependent cell-cell interactions obscures its cellular underpinnings. Here, through a series of genetic rescues in a murine model of brain malformation, the cellular mechanisms underlying this antagonism are uncovered. The expanded apical domain caused by the absence of the basal complex protein, Lethal Giant Larvae (Lgl)1 initiates subsequent cellular defects, including mislocalized adherens junctions and ectopic proliferation. Concurrent genetic removal of apical polarity complex, Crb1/2, abrogates cellular defects and heterotopia, evidence of their pivotal role. Gene expression, cell cycle, and rescue experiments revealed that antagonistic interactions converge onto Hippo-Yap signaling-dependent cell cycle kinetics. Our study highlights the fundamental antagonism of polarity proteins in preventing epithelial disruption and ectopic mitogenic activity.

Apicobasal polarity, a fundamental feature of epithelial cells, provides structural compartmentalization, accommodating functional and mechanical requirements of the cell^1^. Polarity is established and maintained by antagonizing evolutionary complexes, including the basal complex (Lgl, Dlg, Scribble) and the apical complex (Crbs, Pals1, MPDZ, and Par3, Par6, aPKC)^2^ (Fig. 1a). Antagonistic interactions between the apical and basal complexes are initiated by aPKC-mediated phosphorylation, excluding Lgl1 from the apical domain. Physical interactions among the complexes subsequently direct their localized membrane association, segregating them to basal and apical territories^3^. Known mutations in these complex genes cause developmental disorders, including cortical malformations^4,5^. As the cerebral cortex is our center of cognition, capable of higher-order processing, cortical malformations underly intellectual and neurological deficits. In the developing cerebral cortex, stem cell-like neural progenitors termed apical radial glia (aRG) maintain apicobasal polarity, which is crucial for structural integrity and for controlling the number of neurons generated^4,6–8^. Inheritance of apical polarity complex proteins after cell division is associated with self-renewed progenitor fate in other contexts^9,10^, but their role in cortical progenitor fate remains unclear. Overall, our understanding remains limited in how the antagonistic interactions between apical and basal complexes instruct cell fate and polarized structure during cerebral cortex development.

Here, using a genetic rescue strategy, we uncovered an antagonistic relationship among polarity complex proteins in cortical progenitor cells that is essential for both cell junction formation and controlled proliferation. We identified antagonism as a critical upstream regulator of Hippo-Yap signaling, which is responsible for the overproduction of progenitors and neuronal outputs observed in conditions such as periventricular heterotopia. Our study provides a fundamental mechanistic understanding of how the antagonistic interaction between apical and basal polarity complex proteins prevents abnormal mitogenic activation that leads to brain malformation.

## Apical domain expansion in Lgl1 mutants causes junction disruption and heterotopia.

One of the most striking brain malformations has been observed in Lgl1 basal polarity complex gene mutations, including the accumulation of excessive neurons along the ventricles, characteristic of periventricular heterotopia^4^. However, despite its fundamental importance, it is unknown whether this resulted from the loss of antagonism. To determine whether the mechanism initiating this alteration is owing to the failure to antagonize the activity of the apical polarity complex, we first characterized overall polarity after deleting *Lgl1* in the dorsal cortex with Emx1-Cre (hereafter referred to as *Lgl1* cKO)^11^. *Lgl1* cKO causes severe heterotopia apparent underneath the ventricular lining at embryonic (E) 12.5 and massively grows over time (Extended Data Fig. 1) ^4,7,12^. There is also an increase in cell death, in addition to a lengthened ventricular surface (Extended Data Fig. 2). To detect the initial cellular phenotype prior to heterotopia formation at E12.5, we examined E11.5 cortices to reveal early cellular changes that initiate the phenotype. In line with antagonism between basal and apical polarity complexes, the *Lgl1* cKO revealed an expansion of the apical polarity complex proteins as indicated by Pals1, Crbs, and Par3 localization (Fig. 1b–e, Extended Data Fig. 3a-b). This confirms an evolutionarily conserved function of Lgl1 in repressing the apical domain. To assess whether the expanded apical domain in the *Lgl1* cKO is responsible for periventricular heterotopia formation, we deleted apical polarity complex proteins. Unlike other severe apical polarity complex mutants,^13^ the absence of apparent neurogenesis defects in Crb1/2 double mutants enabled us to delete Crb1/Crb2 and generated a triple KO line (hereafter referred to as *Crb1/2/Lgl1* tcKO) (Fig. 1f). Remarkably, the *Crb1/2/Lgl1* tcKO had significantly less heterotopia compared to the *Lgl1* cKO alone at both E14.5 and postnatal day (P) 5. Cellular output of both early and late born neurons was also largely restored at P21 (Extended Data Fig. 4a-e). Also, a striking rescue of hippocampus developmental defects was observed in tcKO (Fig. 1i, Extended Data Fig. 4f-i). These remarkable rescues clearly demonstrate that Lgl1 normally functions to repress the apical domain, which is necessary to prevent periventricular heterotopia.

Previous studies suggest that the position of the adherens junction (AJ) is determined by a tug-of-war between the activities of basal and apical polarity complex proteins^14^. Furthermore, mispositioned AJs are observed in Lgl1 mutants and are claimed to be the major driver of ectopic proliferation, as previously shown in other junction mutants^15,16^. Remarkably, AJs marked by N-cadherin or β-catenin, which were ectopically located in the *Lgl1* KO (Fig. 1l), were completely restored at the ventricular surface in the *Crb1/2/Lgl1* tcKO at E14.5. This clear-cut genetic evidence uncovered that AJ disruption and heterotopia formation resulted directly from apical domain expansion owing to the deficiency of counteracting basal activity (Fig. 1k–m, Extended Data Fig. 3c-d). Because of the necessity of Crb1/2 for maintaining AJ, tcKO showed no AJ at E16.5 late neurogenesis, similar to Crb1/2 dcKO^13^, underscoring the importance of developmental timing to obtain a definite rescue effect (Extended Data Fig. 5).

## The balance of apicobasal polarity prevents Yap activation.

To mechanistically understand how expansion of the apical domain drives overproliferation, we performed bulk RNA sequencing from cortices isolated at E14.5 on control, *Lgl1* cKO, and *Crb1/2/Lgl1* tcKO. As heterotopia is largely rescued, we reasoned that mitogenic signaling dependent on antagonistic interactions should be upregulated in *Lgl1* cKO compared to control and tcKO. In fact, there are 65 genes commonly upregulated in the *Lgl1* cKO when compared to both control and *Crb1/2/Lgl1* tcKO, suggesting these genes are strong candidates contributing to rescue effects of *Crb1/Crb2* deletion (Fig. 2f). In line with the restored cortex, there are minimal number of differentially expressing genes in *Crb1/2/Lgl1* tcKO compared to control (Fig. 2b–e). Considering previous associations between polarity proteins and Notch signaling, we anticipated rescue hits relating to Notch^7^. However, out of the 65 genes, many were directly involved in Hippo-Yap signaling and/or downstream effectors (Fig. 2g). GSEA analysis showed significant enrichment in Yap signaling, rather than Notch (Fig. 2h–i, Extended Data Fig. 6). This suggests that the molecular changes responsible for the *Crb1/2/Lgl1* tcKO genetic rescue are acting through modulating Yap/Taz activity. Collectively, these data indicate that expansion of the apical domain activates Yap signaling, which can be prevented by Crb1/2 deletion.

To date, Hippo-Yap signaling has not been implicated in prior studies of Lgl1 cortical mutants, and the ability of Yap activation to direct cortical progenitor proliferation, leading to excessive neuronal production, is debatable^4,12,17,18^. To gain an unbiased view of molecular changes across different cell populations in *Lgl1* cKO, we performed single-cell RNA sequencing of E15.5 cortices on control and *Lgl1* cKO mice when heterotopia is already prominent. Cells were clustered into nine appropriate groups according to marker genes (Fig. 3a–b, Extended Data Fig. 7). No new cluster molecularly distinct from control was generated by Lgl1 deletion; instead, the notable difference was the increased proportion of proliferating progenitors in the *Lgl1* cKO mice (Fig. 3b, e). In addition, pseudotime analysis revealed more aRG and a lower propensity to progress through differentiated cells such as basal progenitors and neurons (Fig. 3c–d). Cell cycle analysis showed an increased proportion of cells in S-phase in Lgl1 mutants. Consistent with the findings from our bulk RNA sequencing, there was a significant upregulation of Yap target genes in aRG, and these results were confirmed by increased protein expression of Yap target AJUBA in immunostaining and western blot (Fig. 3h–l).

Yap and its paralog WWTR1 (Taz) are transcriptional coregulators largely controlled by a cascade of upstream inhibitory kinases in the Hippo pathway. The kinases phosphorylate and inactivate Yap/Taz in the cytoplasm. When the Hippo kinases are inactive, Yap/Taz translocate to the nucleus and promote the transcription of genes regulating cell proliferation, survival, and fate decision^19^ (Fig. 3m). Yap/Taz are required for cortical progenitor proliferation but only in the later stages of neurogenesis^20^, providing an opportunity to test their requirement in heterotopia formation genetically without complicating their normal function. To determine the contribution of Yap/Taz activation in heterotopia formation, we genetically removed *Yap/Taz* in addition to *Lgl1* (hereafter referred to as *Yap/Taz/Lgl1* tcKO) (Fig. 3n). At E14.5, there is significantly less heterotopia seen in the *Yap/Taz/Lgl1* tcKO compared to the *Lgl1* cKO. Additionally, the cellular output of both early and late born neurons, as well as hippocampal development, was also largely restored at P21 (Extended Data Fig. 8). These data establish that Yap/Taz are important drivers of heterotopia formation.

## Antagonistic interaction of polarity complex regulates cell cycle through Yap/Taz signaling.

As previously mentioned, Yap/Taz are known regulators of cell cycle progression^21–25^. Considering this, in addition to the changes we see in cell cycle phase distribution in our scRNA seq data, we reasoned that Yap/Taz activation may be driving heterotopia through altered cell cycle dynamics. It was previously found that there was reduced cell cycle withdrawal in Lgl1 mutants and no change in cell cycle length^7^. However, this was an indirect estimate of cell-cycle length and did not address the individual cell-cycle phases within aRG. We therefore sought to determine more precisely how Lgl1 loss impacts the cell cycle kinetics of aRG by using cumulative labeling with the nucleotide analog bromodeoxyuridine (BrdU) (Fig. 4a–b). Consistent with our scRNA-seq results, the S fraction (T_S_/T_C_) was higher in the *Lgl1* cKO. While the time encompassing G2M+G1 (T_G_) for control was 12.5 hours, it was reduced to 9.5 hours in the *Lgl1* cKO cortices (Fig. 4c–d). From this, we determined that total cell cycle length (T_C_) was 20.16 hours in control and 18.27 hours in *Lgl1* cKO, and that S phase length (T_S_) was 7.66 hours in control and 8.77 hours in *Lgl1* cKO (Fig. 4c–d). Importantly, a longer T_S_ in neural progenitors corresponds with a more proliferative/self-renewal fate, rather than a differentiative/neurogenic fate. Together, our findings align with previous evidence supporting Yap/Taz’s role in promoting the G1/S transition to contribute to proliferation^22,25^.

Taking advantage of the measurable high S-fraction in *Lgl1* cKO, we tested whether cell cycle changes were recovered by removal of Crb1/2 or Yap/Taz. We assessed the S fraction in the tcKO lines by performing a 30-minute BrdU pulse at E14.5 and E16.5 (Fig. 4e). In both the *Crb1/2/Lgl1* tcKO and *Yap/Taz/Lgl1* tcKO the increased S-fraction observed in the *Lgl1* cKO was returned to normal (Fig. 4f, h–i, k, m–n). Thus, expanded apical domain and downstream activation of Yap/Taz are responsible for increased proliferation through modifications of the cell cycle.

In addition, we tested whether Crb1/2 or Yap/Taz deletions can also rescue the previously identified^7^ excessive proliferative cell division in *Lgl1* cKO. We measured the fraction of aRG labeled by Sox9 among total cells that underwent one cell division by BrdU labeling progenitors 24 hrs prior. Consistent with more proliferative divisions, a higher fraction of BrdU+/Sox9 + among total BrdU+ cells in *Lgl1* cKO mice indicates that aRG undergoes more self-renewing/proliferative divisions^7,26^. This altered progenitor division mode is completely abolished in the *Yap/Taz/Lgl1* tcKO as well as in the *Crb1/2/Lgl1* tcKO (Fig. 4g, j, l, o). Together, these data underscore a failed antagonistic polarity interaction, leading to Yap/Taz-dependent cellular mechanisms that drive heterotopia in the absence of Lgl1.

## Apical expansion drives Yap/Taz activation through nuclear translocation.

Yap and its orthologs are known as cell polarity sensors in other epithelial cells; however, our result is distinct from those reported. For instance, skin basal cells lacking an apical domain exhibit strong Yap activity^27^. Similarly, Crb proteins are well-established upstream inhibitors of Yap by promoting the Hippo pathway^28,29^. Then, how does apical expansion lead to Yap/Taz activation during cortical development? To ask whether apical expansion increases Yap activity via nuclear translocation, a key regulatory step, we examined the proportion of aRG cells with nuclear Yap. An increased fraction of Sox9 + Yap+ among Sox9 + aRGs is observed in *Lgl1* cKO, which was returned to normal in the *Crb1/2/Lgl1* tcKO (Fig. 5a, c). We further confirmed Yap activation by increased levels of Yap target AJUBA in the *Lgl1* cKO and returned to the control level in the *Crb1/2/Lgl1* tcKO (Fig. 5b, d). A similar observation is made for other downstream target proteins, such as Ccn2, by Western blot analysis (Fig. 5h, l).

We next examined the mechanisms underlying Yap nuclear translocation induced by apical domain expansion. We first assessed the phosphorylation site of Yap responsible for cytoplasmic retention (S112 in mouse). Notably, the ratio of S112 phosphorylated Yap to total Yap was substantially reduced in the *Lgl1* cKO and normal in the *Crb1/2/Lgl1* tcKO. This shows that Crb1/2 removal restores negative regulation of Lats1/2 or other kinases through phosphorylation-mediated nuclear translocation (Fig. 5h, j). Interestingly, the level of Nf2, known to play an important role in junction assembly, Yap regulation, and Lats1/2 activation, was significantly increased in the *Crb1/2/Lgl1* tcKO compared to control and *Lgl1* cKO. This result suggests that increased Nf2 may contribute to the rescue effects of reducing Yap activity^30–32^. Together, these results provide evidence that the expansion of the apical domain in *Lgl1* cKO increases Yap/Taz activity by controlling nuclear localization through upstream kinase-dependent phosphorylation.

Finally, we asked through what mechanism apical domain expansion can affect Yap activity through reducing inhibitory phosphorylation. Because mechanotransduction is known to influence the activity of Yap/Taz^33–35^, it is possible that mechanical properties at the ventricular surface are affected by apical domain expansion. It was previously established that increased endfeet surface area of neural progenitors elevates mechanical tension, thereby activating Yap^35^. As another study has shown that Crb overexpression can enlarge the apical surface area in zebra fish retina^36^, we examined the size of apical endfeet in *Lgl1* cKO mice by visualizing concentrated actin at the apical junction near the ventricular surface prior to the onset of heterotopia at E11.5 (Fig. 5e). We found that apical endfeet are significantly wider, by approximately two-fold in *Lgl1* cKO, compared to those of controls (Fig. 5f–g). We observed a complete restoration of apical endfeet size to control levels in the *Crb1/2/Lgl1* tcKO (Fig. 5f–g). As further evidence of apical expansion being upstream of Yap/Taz activation, the *Yap/Taz/Lgl1* tcKO apical endfeet remain enlarged (Extended Data Fig. 9). Similarly, mispositioned AJs were not corrected by Yap/Taz deletion (Extended data Fig. 9). We therefore propose a model in which apical expansion-mediated mechanical tension recruits tension-sensitive junction-associated Lats1/2 inhibitors, such as Ajuba or Nf2, thereby activating Yap through preventing phosphorylation (Fig. 5m). Collectively, our results identify sequential events leading to periventricular heterotopia and highlight how an imbalance in the antagonistic interactions of polarity complex proteins serves as a critical initiator of this severe brain malformation.

## Discussion

Epithelial apicobasal characteristics in cortical progenitors enable them to communicate with neighboring cells via the apical junctions and receive mitogenic signals. However, it is unclear how apicobasal polarity contributes to the regulation of proliferation and progenitor fate. While previous studies have focused on the inheritance of apical or basal processes in relation to mitotic spindle orientation^37,38^, our study provides new cellular and molecular insights into the impact of a fine-tuned balance between apical and basal polarities in cortical progenitor cells. Contrary to the previous works on isolated effects of apical or basal complex proteins on Hippo-Yap signaling^39–42^, distinct from our findings, our study underscores the critical importance of a balance between them, which is necessary to prevent overproliferation by activating Yap/Taz.

During development, cellular contexts evolve at a rapid pace, making it difficult to pinpoint initiating insults in developmental disorders. It is especially difficult to formulate genetic rescue experiments, which are often complicated by other indirect functions. One of the key advantages of our study is the availability of genetic models for rescue experiments, such as Crb1/2 and Yap/Taz mutants, that have no overt defects^13,20^ at the peak of neurogenesis, when Lgl1 mutants exhibit prominent heterotopia. At these early developmental points, we observe unambiguous rescue effects and identify the initial events that lead to subsequent cellular abnormalities.

Our study highlights the importance of apical expansion as a causal effect on AJ mispositioning. Previous studies have demonstrated that physical interaction between Lgl1 and N-cadherin is crucial for preventing AJ formation in the basal region, suggesting that Lgl1 promotes the endocytic removal of N-cadherin^4^. At the apical side, aPKC phosphorylates and inactivates Lgl1, allowing N-cadherin to contribute to AJ formation at the border of the apical domain. It is plausible that, in Lgl1 mutants, because Lgl1 is no longer present to prevent basal targeting of N-cadherin, AJs form at the basal side. However, if apical complex function is simply to remove Lgl1 at the apical domain, N-cadherin would not be returned to the apical side through Crb1/2 elimination. Our data indicates that ectopic N-cadherin resulted from an expanded apical polarity complex, supporting the active role of this complex in ectopic AJ formation. Furthermore, because Yap activation is also restored when AJ is rescued in the tcKO, AJ mislocalization may also trigger Yap activation. Consistent with this idea, the deletion of cytoskeletal regulators and AJ components, including N-cadherin, Afadin, and RhoA, in cortical progenitors causes heterotopia ^15,16,43^. However, for most of them, Yap activation has not been tested, making it an exciting future research topic to determine how broadly applicable Yap activation is to junction disruption-mediated heterotopia.

Many previous studies have shown that tension promotes cytoskeletal contraction, an elevated membrane potential, and cell mass, thereby activating Yap^34,44^. Our study suggests that increased tension at the apical junction, due to apical endfeet widening, recruits tension-dependent Yap regulators, such as Ajuba, to bind and inhibit Lats activity, as shown in the Fly epithelium^45^. In turn, the lack of Lats-mediated phosphorylation promotes the nuclear accumulation and activation of Yap/Taz, driving overproliferation. Interestingly, our transcriptomic study shows that Ajuba is a prominent downstream effector upregulated by Lgl1 loss but normalized by Crb1/2 loss. It is likely that increasing Ajuba provides positive feedback mechanisms that enhance Lats1/2 inhibition, thereby further facilitating Yap/Taz activation. Furthermore, the reduction of Nf2, a junction-associated protein shown to bind Crb proteins, likely also contributes to Yap/Taz activation. Nf2 is a well-established Yap inhibitor through multiple regulatory mechanisms, so it is possible that Crb expansion or junction mispositioning reduces Nf2 levels by promoting Nf2 degradation^30,31,46^. Importantly, Nf2 level is returned to normal in *Crb1/2/Lgl1* tcKO, suggesting that the decreased Nf2 level in Lgl1 mutant is Crb1/2 dependent. Further research on these upstream regulators of Yap/Taz at the junction would yield a better understanding of their regulatory role in Yap-dependent mitogenic control.

In humans, periventricular heterotopia is a genetically heterogeneous condition, with many genes associated with malformation^47^. Loss of function of LLGL1, the human homolog of Lgl1, has yet to be attributed to human cases of PH. However, the human condition Smith-Magenis syndrome (SMS) is associated with a 17p11.2 deletion that spans the LLGL1 gene. SMS is characterized by intellectual disability, dysmorphic features, and short stature. While structural brain anomalies are not a hallmark of this syndrome, neuroblastoma and periventricular nodules have been reported in some cases^48,49^. Our work demonstrating PH formation in the Lgl1 mutant, along with the known tumor-suppressor role of Lgl, suggests that decreased LLGL1 may, at least in part, contribute to the PH in these patients. Interestingly, although mouse models have shown PH with Yap activation, cortical organoid experiments using FAT4 and DCHS, known causative genes of PH, reveal that their PH formation is not due to hyperproliferation but rather to molecular changes in neurons, representing a premature, developmentally misprojecting abnormal state ^21,22^. It would be valuable to have a human model of LLGL1 to address these species differences in PH formation.

Our study may offer new insights to other pathogenic mechanisms such as cancer, as aberrations in cell polarity are a hallmark of epithelial cancer^50^. Consequently, accumulating evidence has shown that loss of LLGL1 is implicated in various forms of cancer and is associated with cancer progression and poor prognosis^51–54^. While LLGL1 loss-of-function appears to be cell-type-specific in its role in cancer progression, most studies suggest that it contributes to proliferative capacity and self-renewal propensity. Our study may provide an indication to how Yap is activated in these conditions. It will be worthwhile determining whether the underlying mechanisms described here, involving apical domain expansion followed by Yap activation, also contribute to cancer progression in these patients. Yap inhibitors are currently being explored as therapeutic targets for certain cancers, and apical polarity complex protein, aPKC, inhibitors have been applied to preclinical cancer models^55,56^. Our work may suggest their utility in other cancers associated with Yap activation and cell polarity disruption.

## Methods

### Mice

Animal experiments were performed in accordance with the guidelines of the Institutional Animal Care and Use Committee of Lewis Katz School of Medicine at Temple University (Protocol 5059). Food and water were provided *ad libitum* and animals were kept on a 12-hour light/dark cycle. Animals were kept at temperatures between 70 and 74 °C and humidity between 30% and 70%. For every experiment, 3–8 animals from each mouse line were analyzed. For determination of age of embryos amongst time mating, presence of a copulatory plug in the morning was considered embryonic day 0.5. Sex differences are not a factor in experiments at embryonic timepoints and have no obvious effects on cortical development in these models. Mice with dorsal cortex-specific knockout of Lgl1 (Lgl1 cKO) were obtained by breeding mice carrying a conditional Lgl1flox/flox allele (kindly provided by Dr. Vasioukhin from Fred Hutch Cancer Center) with Emx1-Cre transgenic mice (The Jackson Laboratory, strain number 005628) ^17^. Lgl1^flox/flox^;Emx1-Cre^+/−^ males were crossed with Lgl1^flox/flox^ -females. Mice with dorsal cortex-specific knockout of Yap/Taz/Lgl1 (*Yap/Taz/Lgl1* tcKO) were obtained by breeding mice carrying conditional Yap/Taz ^flox/flox^ allele ^63,64^ with Lgl1^flox/flox^ allele. Yap/Taz/Lgl1^flox/flox^ females were then bred with Yap/Taz/Lgl1^flox/flox^ Emx1-Cre^+/−^ males. Mice with dorsal cortex-specific knockout of Crb1/Crb2/Lgl1 (Crb1/2/Lgl1 tcKO) were obtained by breeding mice carrying conditional Crb1/Crb2 ^flox/flox^ allele with Lgl1^flox/flox^ allele. Crb1/Crb2/Lgl1^flox/flox^ females were then bred with Crb1/Crb2/Lgl1^flox/flox^ Emx1-Cre^+/−^ males. Mice were on mixed backgrounds. Genotyping was performed on toe or tail samples, and all primers are listed in key resources table.

### Histology

Tissue samples were embedded in paraffin and sliced into 7 μm sections. Samples were rehydrated in a series of ethanol and xylene washes and then stained with hematoxylin and eosin (H&E) using standard procedures.

### Single Cell RNA-Sequencing

Genes that were detected in at least 3 cells and cells with a minimum of 200 genes were analyzed with Seurat v5 (adapted from https://github.com/satijalab/seurat . Data was then filtered to include genes represented in more than 2000 cells; the total amount of RNA per cell greater than 500; percent of mitochondrial reads less than 5%; and the percent of ribosomal less than 20%. Doublets were eliminated with DoubletFinder with principal components (PCs) = 1:10, proportion of artificial doublets (pN) = 0.25, and the PC neighborhood size used to compute proportion of artificial nearest neighbors (pK) = 0.09. Data was then normalized using SCTransform with the first 30 PCs, k.param (k)= 50, resolution of 0.5, neighbors= 15, and Leiden algorithm (algo=4). Normalized data was integrated and UMAPs were produced using ggplot2. Findallmarkers was ran on the integrated data to generate a list of markers for each cluster. Clusters were assigned based on common marker genes described in previous papers ^65–67^. A list of differentially expressed genes was generated by comparing conditions and filtering for average log fold of greater than 0.5 and padj of less than 0.05. Monocle3 was used for psueudotime analysis on integrated data to plot the cell density over pseudotime branch points (adapted from https://github.com/cole-trapnell-lab/monocle3). Cell cycle scoring was run using Seurat’s cell cycle scoring function and plotting the proportion of cells to display the cell cycle phase distribution. GSEA analysis was ran with gsea2 and ridge plots were generated to display most enriched pathways. VlnPlot2 was used to generate violin plots for Yap target genes.

### Immunofluorescence

Tissue samples were embedded in paraffin and sliced into 7 μm sections. Samples were rehydrated in a series of xylene and ethanol washes and rinsed in distilled water. Antigen retrieval was performed on sections by boiling samples in a solution of sodium citrate with Tween-20. Samples were then washed in phosphate-buffered saline (PBS). Samples were incubated in primary antibodies with 5% normal goat serum at 4 °C overnight and rinsed again in PBS the next morning. Samples were incubated in secondary antibodies (Alexa Flour 488 anti-mouse, Alexa Fluor 488 anti-chicken, Alexa Fluor 488 anti-rabbit, Alexa Fluor 488 anti-rat, Cy3 anti-rabbit, Cy3 anti-mouse, Alexa Fluor 647 anti-mouse, Alexa Flour 647 anti-rabbit) at a concentration of 1:500 with 5% normal goat serum and 1:1000 Hoechst 33258 at room temperature for 3 h. Samples were washed in PBS three times before being mounted with coverslips using Fluoromount G. Finally, samples were imaged with an IF microscope (SP8, Leica) and image analysis was performed using LAS AF (Leica) and Photoshop (Adobe).

### BrdU Labeling

For 30 min BrdU labeling, 100mg/kg of BrdU in sterile PBS was intraperitoneally injected into E14.5 mice. Embryos were collected for 30 minutes post BrdU injection.

For 24-hour BrdU labeling, 100mg/kg of BrdU in sterile PBS was intraperitoneally injected into E13.5 mice. Embryos were collected exactly 24 hours post BrdU injection.

For cumulative BrdU labeling, 50mg/kg of BrdU in sterile PBS was intraperitoneally injected into E14.5 mice, repeated at 3-hour intervals up to 15 hours. The pregnant mice were collected at the intended time points. After embedding and sectioning samples, slides were stained with BrdU and Sox9 to label all aRG in S phase.

### Analysis of Cell-cycle Parameters

All aRG cells were labeled by Sox9^+^ and proliferative aRG cells were labeled by BrdU^+^Sox9^+^ cells. Cells were manually counted and the proportions of BrdU^+^Sox9^+^ / total Sox9^+^ were plotted and used to determine the following cell cycle parameters:

**Table:** 

Wild Type	Lgl1 cKO
T_G_= 12.5 hours	T_G_= 9.5 hours
T_S_/T_C_= 0.38 hours	T_S_/T_C_= 0.48 hours
T_S_= 0.38 T_C_	T_S_= 0.48 T_C_
T_S_ +T_G_ = T_C_	T_S_ +T_G_ = T_C_
0.38 T_C_ + 12.5 = T_C_	0.48 T_C_ + 9.5 = T_C_
12.5 = T_C_ − 0.38 T_C_	9.5 = T_C_ − 0.48 T_C_
12.5 = 0.62 T_C_	9.5 = 0.52 T_C_
**T_C_ = 20.16 hours**	**T_C_ = 18.27 hours**
T_S_/T_C_= 0.38 hours	T_S_/T_C_= 0.48 hours
T_S_/20.16 = 0.38 hours	T_S_/18.27 = 0.48 hours
**T_S_= 7.66 hours**	**T_S_= 8.77 hours**

### Apical Explant Isolation and Staining

E11 and E12 brains were collected and fixed overnight in 4% PFA. Following overnight fixation, the dorsal cortex of each hemisphere was dissected in cold PBS. After dorsal cortex isolation the samples were fixed again in 4% PFA for 10 minutes at room temperature. Samples were then washed two times (5 minutes each) with PBS while shaking. Antigen retrieval was then performed by adding 1% SDS for 5 minutes at RT. Samples were then washed again two times (5 minutes each) with PBS while shaking. Next samples were permeabilized in 0.1% Triton X-100 for 20 minutes. Samples were then washed again two times (5 minutes each) with PBS while shaking. Primary antibody was added and kept overnight at 4C. On the second day samples were washed two times (5 minutes each) with PBS while shaking. The secondary antibody was added for 1 hour at RT. Samples were then washed two times (5 minutes each) with PBS while shaking. Phalloidin solution (1X in diluted PBS) was then added to each sample for 30 minutes at RT, followed by Hoechst for 10 minutes. Samples were washed again two times (5 minutes each) with PBS while shaking. Samples were then mounted with the apical/ventricular surface facing upwards and the pia side faced down on the slide. Orientation was visualized under an Evos microscope to see RFP signal outlining the blood vessels within the pia.

### 3D reconstruction with Imaris Software

After mounting apical explant samples, they were imaged on a Leica SP8 IF microscope. The apical surface was found by focusing on the mitotic cells with Hoechst staining and then focusing slightly more apical to find the smallest apical endfeet with phalloidin staining. Once at the apical surface, z-stack images were taken at 0.50 μm thickness with the same number of stacks per sample for comparison. For 3D reconstruction with Imaris, the Leica z stack files were converted to Imaris files and analyzed with Imaris software. Images were all cropped to the same parameters. Using the surface tool, select only the channel of interest and adjust the threshold and voxels to match each sample. The color and material to visualize each channel can be selected for better appearance. Snapshots were then taken with the same magnification of each sample. To quantify the basal expansion of Pals1 in the *Lgl1* cKO vs control, the total volume of the Pals1 channel was derived from the 3D reconstruction .

### Bulk RNA-sequencing

Fox Chase Cancer Center (FCCC) Genomic Facility isolated for RNA from cortices collected at E14.5 and sequencing was conducted using Illumina’s HighSeq 2500. Data was processed through Illumina and referenced mapped to the Mus musculus (UCSC mm10) genome using Cufflinks Assembly & DE BaseSpace Workflow (2.1.0), Isis analysis software (2.6.25.27), STAR aligner (STAR_2.5.0b), BEDTools (2.17.0), Cufflinks (2.2.1), BLAST (2.2.26+). Raw gene counts were extracted from Illumina and analyzed in RStudio. Count files from each sample were merged and analyzed with DESeq2. Pheatmap and ggplot2 were used to generate the figures presented.

### Western Blot Analysis

Dorsal cortices were dissected on embryonic day 14 to prepare protein lysates. Protein lysates were homogenized in ice-chilled cell lysis buffer (20mM Tris-HCL (pH 7.5), 150mM NaCl, 1mM Na2EDTA, 1mM EGTA, 1% Triton, 2.5 mM sodium pyrophosphate, 1mM beta-glycerophosphate, 1mM Na3VO4, and 1μg/ml leupeptin) containing 1% Protease Inhibitor Cocktail P8340 (Sigma). Using an SDS-PAGE gel, protein lysates were separated and transferred to a polyvinylidene difluoride membrane. The membrane was blocked with 5% nonfat dry milk, and primary antibodies were applied at 4°C overnight. The primary antibodies included Yap/Taz (1:1000; Abcam), pYap (1:1000; Cell Signaling), NF2 (1:1000), CTGF (1:1000; Cell Signaling), Taz (1000; Cell signaling), and GAPDH (1:5000; Cell Signaling). Expression signals were detected by chemiluminescence (ECL Kit; GE Healthcare).

### Antibodies

Antibodies used: BrdU (1:500), Sox9 (1:500), N-cadherin (1:500), MPP5/Pals1 (1:500), Ajuba (1:500), Yap/Taz (1:500), p-Yap (1:500), panCrb (1:500), Beta-catenin (1:500), Cux1 (1:500), Cleaved Caspase-3 (1:500), CTIP2 (1:500), rH2AX (1:500), Pan Crb (1:500), Notch1 (1:500), Cleaved notch 1 (1:500).

## Supplementary Material

This is a list of supplementary files associated with this preprint. Click to download.

- ExtendedDataFigures.docx

## Figures and Tables

**Figure 1:**
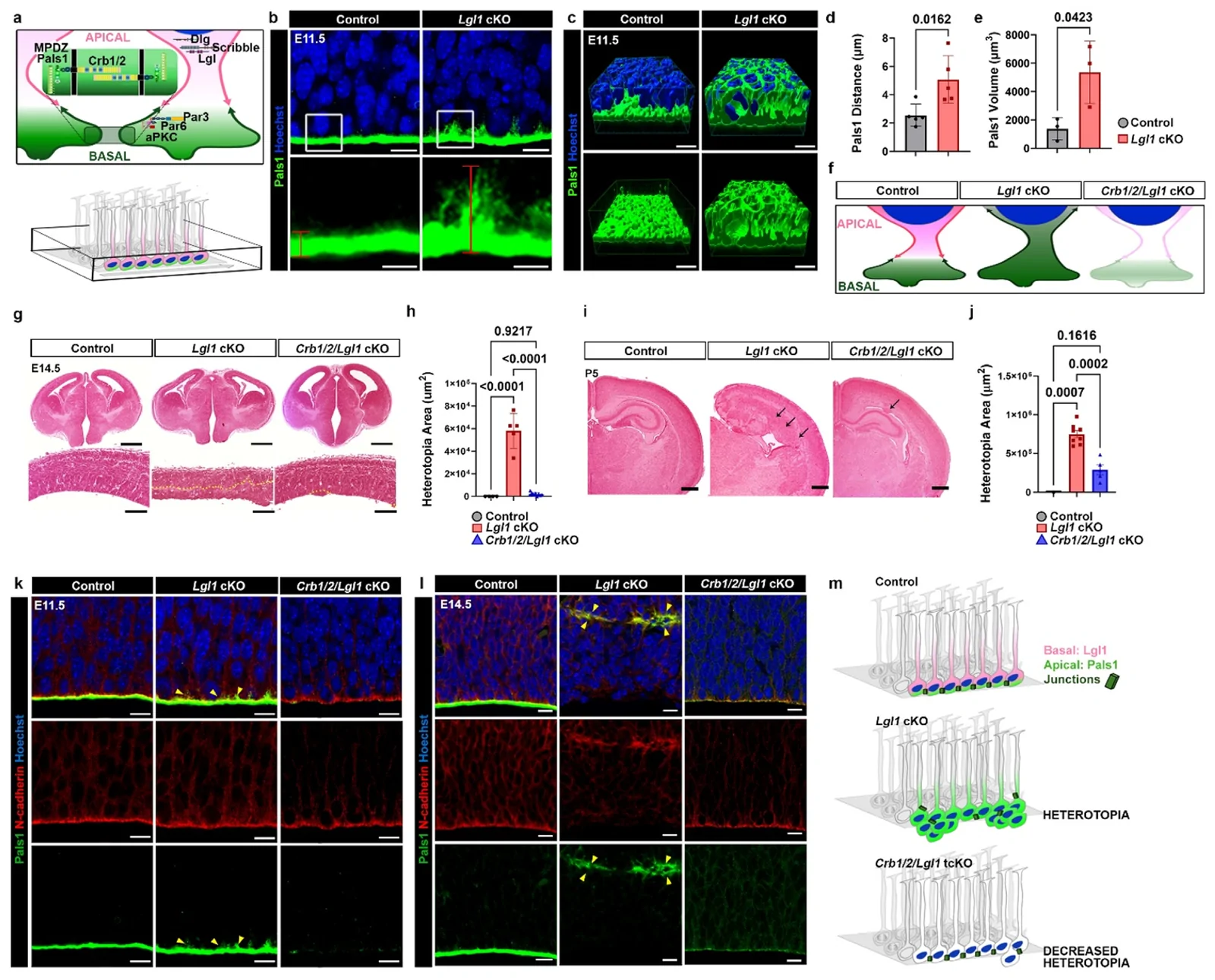
Apical complex expansion drives disrupted junctions and periventricular heterotopia in *Lgl1* cKO mice. **a,** Schematic of apicobasal polarity complexes and their localization in neural progenitors along the ventricular surface. **b,** Representative IF images of Control and *Lgl1* cKO at E11.5 with Pals1. Scale bars (top), 10μm. Scale bars (bottom, magnified), 3.5μm. **c,** Imaris reconstruction of E11.5 Control and *Lgl1* cKO explant samples. Scale bar, 10μm. **d,** Quantification of **(a)**. Each point represents a different sample where the average distance of Pals1 localization (repeatedly measured from most apical point to most basal point) along an 84.5μm length (every 1.1 μm) of the cortex. Red line represents one of these measurements along the cortex. Control n=5; *Lgl1* cKO n=5. **e,** Quantification of Pals1 IF signal volume in **(c)**. **f,** Schematic of apical domain expansion following cKO of *Lgl1* and further model of deleting *Crb1/Crb2* in addition to *Lgl1*. **g,** H&E staining of cortical sections from E14.5 Control, *Lgl1* cKO, and *Crb1/2/Lgl1* tcKO brains. Scale bar top, 500μm. Scale bar bottom, 50μm. **h,** Heterotopia quantification of **(g)**. Control n=4; *Lgl1* cKO n=5; *Crb1/2/Lgl1* tcKO n=9. **i,** H&E staining of cortical sections from P5 Control. *Lgl1* cKO, and *Crb1/2/Lgl1* cKO. *Lgl1* cKO, and *Crb1/2/Lgl1* tcKO brains. Scale bar top, 500 μm. Scale bar bottom, 50μm. **j,** Heterotopia quantification of **(i)**. Control n=1; *Lgl1* cKO n=8; *Crb1/2/Lgl1* tcKO n=5. **k,** Representative IF images of E11.5 Control, *Lgl1* cKO, and *Crb1/2/Lgl1*tcKO with N-cadherin/Pals1 immunostaining. **l,** Representative IF images of E14.5 Control, *Lgl1* cKO, and *Crb1/2/Lgl1* tcKO with N-cadherin/Pals1. **m,** Schematic of apical domain expansion driving heterotopia in *Lgl1* cKO with concurrent KO of Crb1/Crb2 restoring normal development. Each data point (n) represents 1-2 sections per sample. All data bars presented as the mean ± s.e.m. Unpaired t-test (**d-e**); One-way ANOVA with Tukey’s multiple comparison test (**h, j**). *n* represents the number of independent animals.

**Figure 2:**
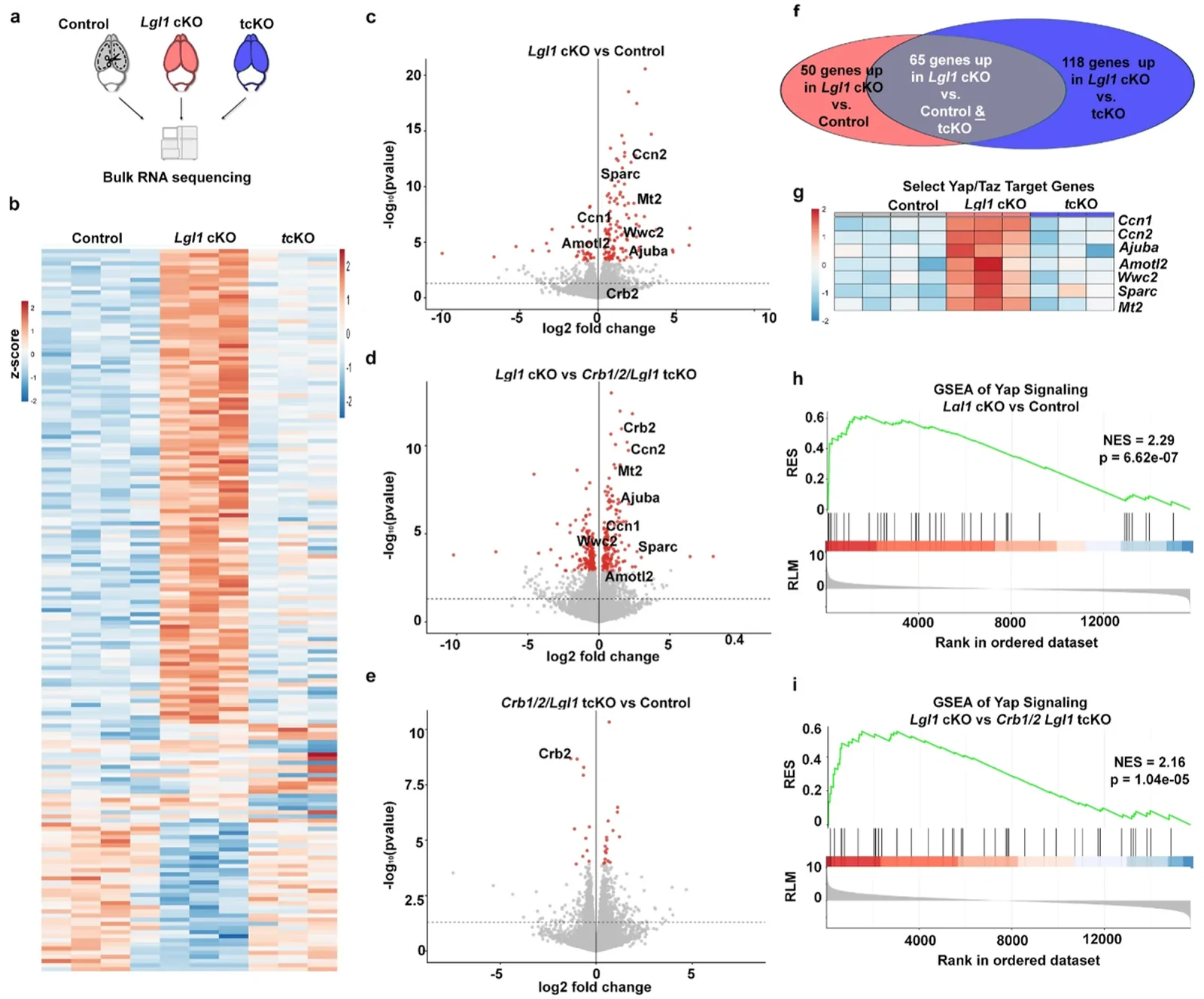
Yap target genes mediate the rescue effect of Crb1/2/Lgl1 tcKO. **a,** Schematic of bulk RNA sequencing workflow. **b,** Heatmap of all differentially expressed genes in *Lgl1* cKO vs Control and *Crb1/Crb2/Lgl1* tcKO vs control. Control n=4, *Lgl1* cKO n=3, *Crb1/2/Lgl1* tcKO n=3. Hierarchical ordering is based on z-score. All genes displayed have padj<0.05. **c,** Volcano plot of *Lgl1* cKO vs Control. Red dots indicate p < 0.05. Grey dots did not reach significance. Select genes labeled. **d,** Volcano plot of *Lgl1* cKO vs *Crb1/Crb2/Lgl1* tcKO. Red dots indicate p < 0.05. Grey dots did not reach significance. Select genes labeled. **e,** Volcano plot of *Crb1/2/Lgl1* tcKO vs Control. Red dots indicate p < 0.05. Grey dots did not reach significance. Select genes labeled. **f,** Venn diagram depiction of all genes upregulated in *Lgl1* cKO compared to Control (left pink circle;115 total) and *Lgl1* cKO compared to *Crb1/2/Lgl1* tcKO (right blue circle; 183 total). Overlapping in the center in purple are genes commonly upregulated in *Lgl1* cKO compared to both Control and in *Crb1/2/Lgl1* tcKO. All genes noted in Venn diagram have log2FC > 0 and padj < 0.05. **g,** Targeted heatmap of Yap target genes *Amotl2, Sparc, Ajuba, Ccn2, Wwc2, Mt2, Ccn1* across all three conditions. **h,** Gene set enrichment analysis (GSEA) of conserved Yap signature genes in *Lgl1* cKO vs Control. Red (left peak) indicates *Lgl1* cKO and blue (right peak) indicates control. **i,** GSEA of conserved Yap signature genes in *Lgl1* cKO vs. C*rb1/2/Lgl1* tcKO. Red (left peak) indicates *Lgl1* cKO and blue (right peak) indicates *Crb1/2/Lgl1* tcKO. RES= Running enrichment score; RLM= Ranked list metric. NES= normalized enrichment score. n represents the number of independent animals.

**Figure 3:**
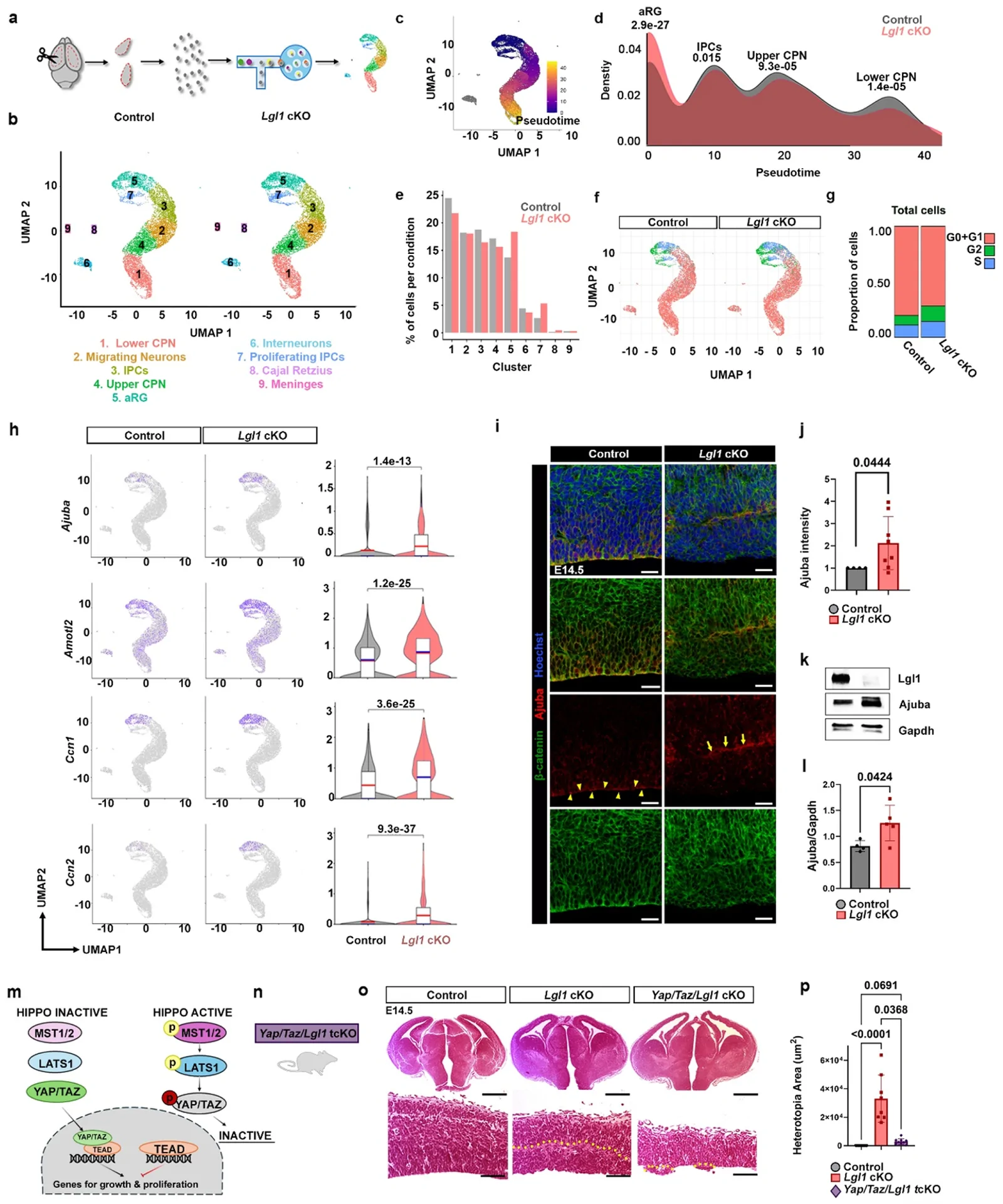
Increased Yap activity in aRG promotes periventricular heterotopia in *Lgl1* cKO mice. **a,** Schematic of scRNA sequencing. **b,** Uniform manifold approximation and projection (UMAP) of Control and *Lgl1* cKO with corresponding cluster identities. Abbreviations: CPN-Callosal Projection Neurons; IPCs-Intermediate Progenitor Cells; aRG- apical Radial Glia. **c,** Pseudotime projection onto UMAP. **d,** Monocle pseudotime projection of Control and *Lgl1* cKO with peaks representing aRG, IPCs, Upper CPN, Lower CPN. **e,** Percentage of cells making up each cluster. **f,** Cell cycle phase projection onto UMAP. **g,** Cell cycle phase distribution. **h,** UMAP projection and violin plots of expression levels of indicated Yap target genes. **i,** Representative IF images of E14.5 sections immunostained with Ajuba and β-catenin. Scale bars, 20mm. **j,** Quantification of Ajuba IF intensity in **(i)**. Control n=4; *Lgl1* cKO n=8. Variation between slides was accounted for by taking the ratio of Lgl1 cKO Ajuba intensity versus Control Ajuba IF intensity within samples on the same slide and litter. The ratio was then plotted against the normalized value of “1” for Control values. **k,** Western blot of Lgl1 and Ajuba in comparison to Gapdh in *Lgl1* cKO and Control littermates. Control n=4; *Lgl1* cKO n=5. **l,** Quantification of Ajuba protein in **(j)**. **m,** Hippo/Yap signaling pathway schematic. **n,** Schematic of *Yap/Taz/Lgl1* tcKO mouse model. **o,** H&E staining of cortical sections from E14.5 Control, *Lgl1* cKO, and *Yap/Taz/Lgl1* tcKO brains. Yellow dotted line indicates heterotopia area. Scale bar top, 500μm. Scale bar bottom, 50μm. **p,**Quantification of heterotopia area in **(o)**. Control n=7; *Lgl1* cKO n=8; *Yap/Taz/Lgl1* tcKO n=9. Each data point (n) represents 1-2 sections per sample. All data bars presented as the mean ± s.e.m. Fisher’s exact test with Benjamani-Hochberg correction of segmented peaks/valleys **(d)**; Pairwise Wilcoxon ranked sum test with holm-Bonferroni correction **(h)**; Unpaired t-test **(j, l)** One-way ANOVA with Tukey’s multiple comparison test (**p**). *n* represents the number of independent animals.

**Figure 4:**
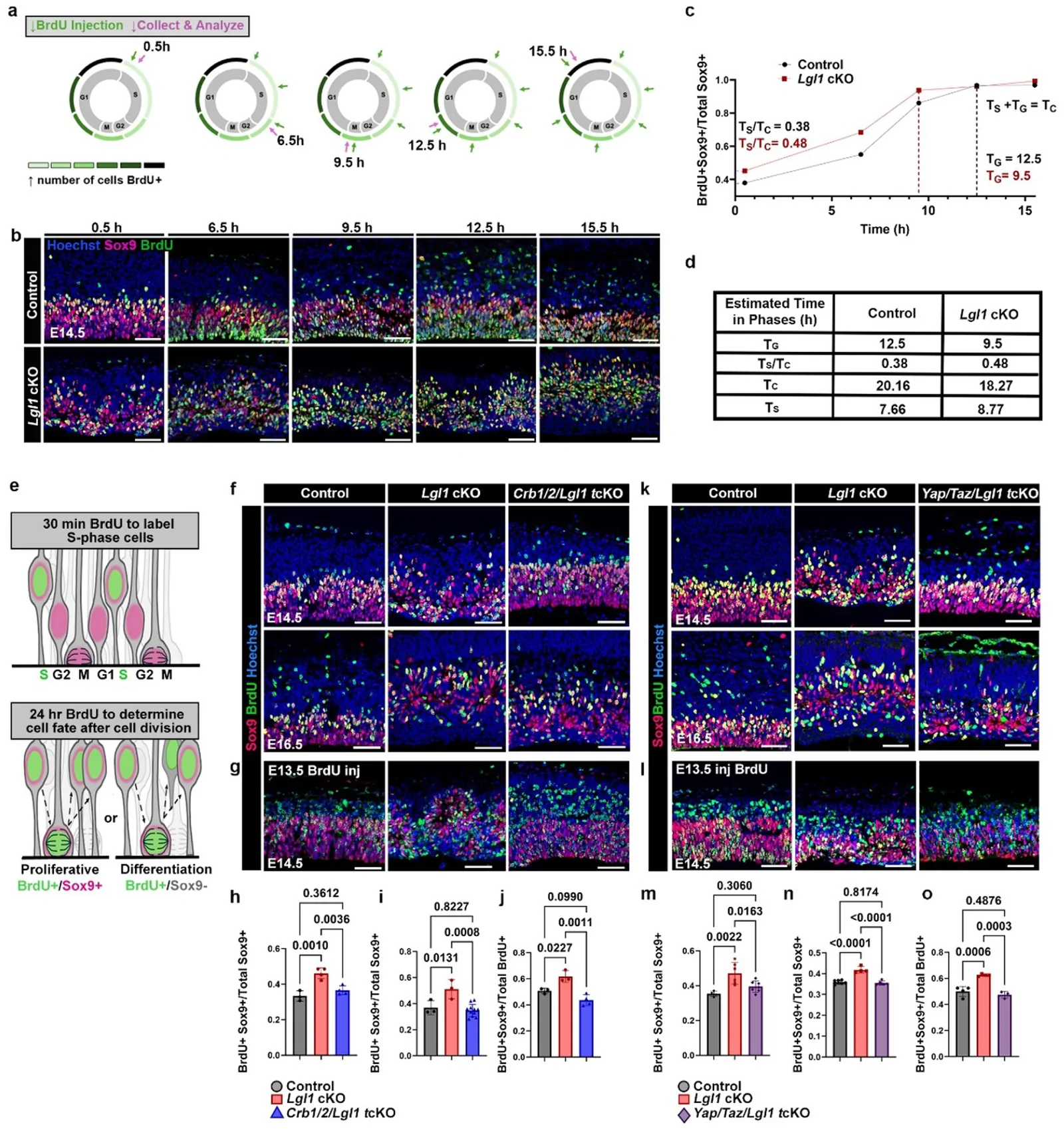
Progenitor defects in *Lgl1* cKO mice are restored after deletion of both *Yap/Taz* and *Crb1/2*. **a,** Schematic of cumulative BrdU experimental design. **b,** Representative confocal images of E14.5 cumulative BrdU labeling at each time point of *Lgl1* cKO and Control littermates. Blue= Hoechst; Red= Sox9; Green= BrdU. Scale bar, 50 mm. **c,** Cumulative BrdU labeling quantification of the proportion of BrdU+/Sox9+/Total Sox9+ cells at each time point (0 hr Control n=4, *Lgl1* cKO n=5, p= 0.0384; 6 hr Control n=3, *Lgl1* cKO n=3, p= 0.0007; 9 hr Control n=5, *Lgl1* cKO n=7, p=0.0077; 12 hr Control n= 4, *Lgl1* cKO n=5, p >0.9999; 15 hr Control n=2, *Lgl1* cKO n=3, p= 0.9811). **d,** Cell cycle parameter calculations. The growth fraction (T_G_) represents the plateau point on the graph, the S-fraction (T_S_/T_C_) represents the number of cells in S phase upon the first injection, or the Y-intercept, T_C_ represents the total cell cycle length, and T_S_ represents the S-phase length. **e,** Schematic of 30 min and 24-hour BrdU labeling used in (f-g; k-m). **f,** Representative IF images of BrdU (0.5-hour labeling) and Sox9 in E14.5 and E16.5 *Lgl1* cKO, *Yap/Taz/Lgl1* tcKO, and their littermate Controls. Scale bars, 50μm. **g,** Representative IF images of BrdU (24-hour labeling) and Sox9 in E14.5 *Lgl1* cKO, *Yap/Taz/Lgl1* tcKO, and their littermate Controls. Scale bars, 50μm **h,** Quantification of the percentage of BrdU+Sox9+ over total number of Sox9+cells E14.5 30-min BrdU in **(f)**. Control n=4, *Lgl1* cKO n=4, *Crb1/2/Lgl1* tcKO n=3. **i,** Quantification of the percentage of BrdU+Sox9+ over total number of Sox9+cells E16.5 30 min BrdU in **(f)**. Control n= 3, *Lgl1* cKO n= 3, *Crb1/2/Lgl1* tcKO n= 11. **j,** Quantification of the percentage of BrdU+Sox9+ over total number of BrdU+ cells 24-hour BrdU in **(g)**. **k,** Representative IF images of BrdU (0.5-hour labeling) and Sox9 in E14.5 and E16.5 *Lgl1* cKO, *Yap/Taz/Lgl1* tcKO, and their littermate Controls. Scale bars, 50μm. **l,** Representative IF images of BrdU (24-hour labeling) and Sox9 in E14.5 *Lgl1* cKO, *Yap/Taz/Lgl1* tcKO, and their littermate Controls. Scale bars, 50μm. **m,** Quantification of the percentage of BrdU+Sox9+ over total number of Sox9+cells for E14.5 in **(k)**. Control n=4; *Lgl1* cKO n=6; *Yap/Taz/Lgl1* tcKO n=7. **n,** Quantification of the percentage of BrdU+Sox9+ over total number of Sox9+cells for E16.5 in **(k)**. Control n=6; *Lgl1* cKO n=4; *Yap/Taz/Lgl1* tcKO n=5. **o,** Quantification of the percentage of BrdU+Sox9+ over total number of BrdU+ cells for E14.5 in **(l)** Control n=4; *Lgl1* cKO n=4; *Yap/Taz/Lgl1* tcKO n=3. Each data point (n) represents 1-2 sections per sample. All data bars presented as the mean ± s.e.m. Sidak’s multiple comparison test **(c)**; One-way ANOVA with Tukey’s multiple comparison test **(h-j, m-o)**. *n* represents the number of independent animals.

**Figure 5:**
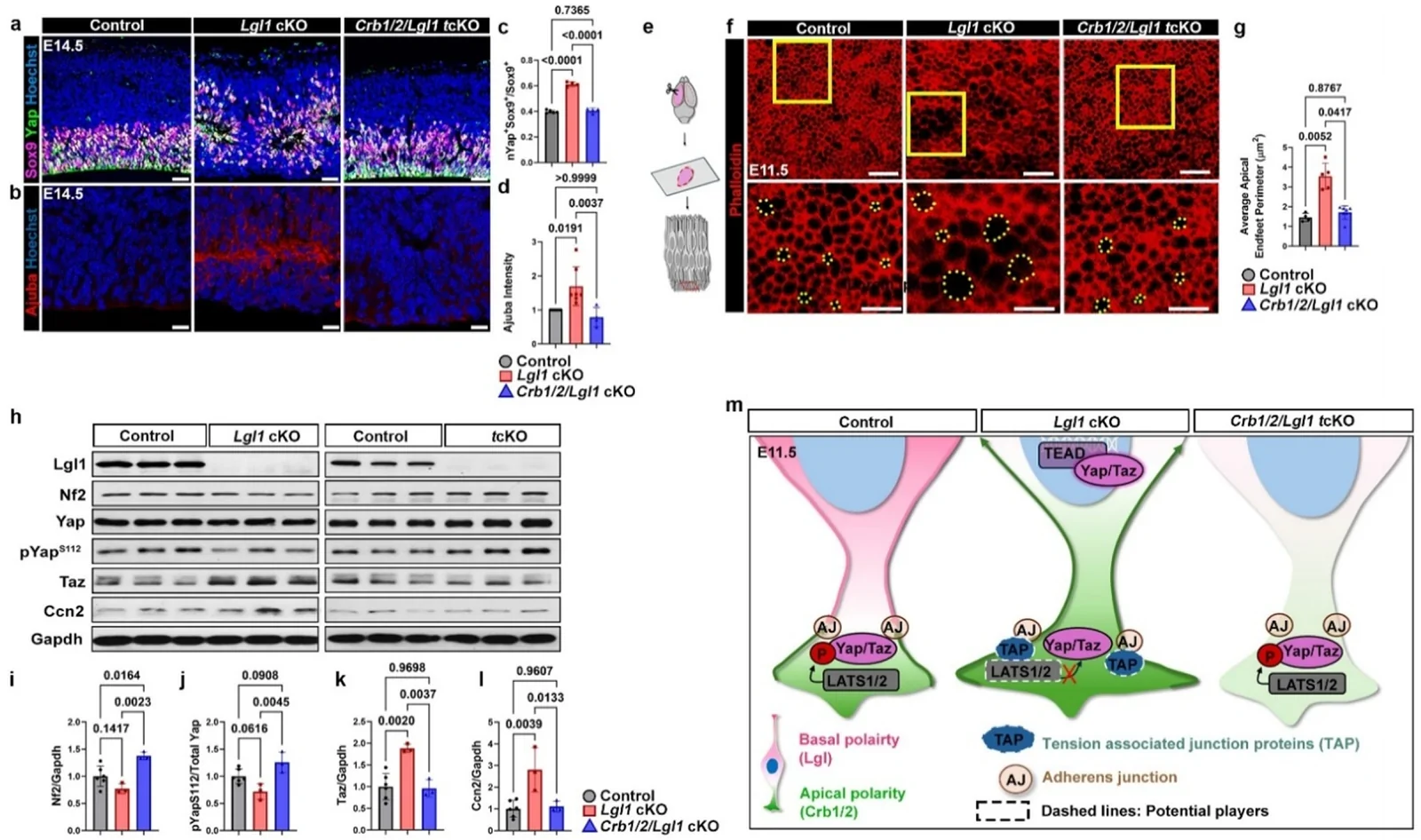
Apical domain expansion promotes Yap activity by reducing inhibitory phosphorylation. **a,** Representative IF images Yap/Sox9 staining of E14.5 cortices in Control, *Lgl1* cKO, and *Crb1/2/Lgl1* tcKO. Scale bars, 25 μm. **b,** Representative IF images Ajuba staining of E14.5 cortices in Control, *Lgl1* cKO, and *Crb1/2/Lgl1* tcKO. Scale bars, 10 μm. **c,** Quantification of Sox9+Yap+/Total Sox9+ in **(a)**. Control n=5; *Lgl1* cKO n=4; *Crb1/2/Lgl1* tcKO n=4. **d,** Quantification of Ajuba IF signal intensity in **(b)**. Control n=5; *Lgl1* cKO n=4; *Crb1/2/Lgl1* tcKO n=4. **e,** Schematic experimental design for apical endfeet whole mount staining. **f,** Representative IF images of apical endfeet staining with phalloidin and quantification of E11 cortices. Scale bar top, 15μm. Scale bar bottom, 7.5μm. **g,** Quantification of apical endfeet size in **(f)**. Control n=4; *Lgl1* cKO n=5; *Crb1/2/Lgl1* tcKO n=7. **h,** Western blot for Lgl1, Nf2, Yap, pYap (phosphorylation site S112), Taz, Ccn2, and Gapdh from E14.5 embryos. **i,** Quantification of Nf2 intensity in **(h)**. n = 3 samples per condition. **j,** Quantification of pYap^**S112**^ intensity in **(h)**. n = 3 samples per condition. **k,** Quantification of Taz intensity in **(h)**. n = 3 samples per condition. **l,** Quantification of Ccn2 intensity in **(h)** n= 3 samples per condition. **m,** Overall working hypothesis. Under normal conditions Lats1/2 phosphorylates and inactivates Yap/Taz. With the removal of Lgl1, the apical domain (Crb1/Crb2) shift basally, leading to a widening of the endfeet. Widening of the endfeet recruits tension associated junction proteins that inactivate Lats1/2, allowing Yap/Taz to translocate to the nucleus. Removal of the apical domain proteins Crb1/2 in addition to Lgl1 prevents widening of the endfeet and downstream activation of Yap/Taz. Each data point (n) represents 1-2 sections per sample. All data bars presented as the mean ± s.e.m. One-way ANOVA with Tukey’s multiple comparison test **(c-d, g, i-l)**. *n* represents the number of independent animals.
